# Correlation between Parental Hostility and Child Self-Control and Aggression

**DOI:** 10.3390/healthcare11172433

**Published:** 2023-08-31

**Authors:** Sun Yee Yoo, Hye Young Ahn

**Affiliations:** 1 Department of Nursing, Gwangju University, Gwangju 61743, Republic of Korea; syyoo@gwangju.ac.kr; 2 College of Nursing, Eulji University, Uijeongbu 11759, Republic of Korea

**Keywords:** parent hostility, child, self-control, aggression

## Abstract

(1) Background: Child aggression is not easily reduced as children grow up, and it is a serious problem that can develop into a life of crime if left unaddressed. (2) Methods: This study was conducted among elementary school children and their parents in C and K provinces and D city. Data were collected through a survey. Pearson’s correlation coefficient was used to analyze parental hostility and each child’s self-control and aggression. (3) Results: Child aggression had a significant negative correlation with self-control and a significant positive correlation with parental hostility. In particular, there was a significant positive correlation between physical aggression and revenge, which are sub-factors of parental hostility. In addition, the children’s self-control was significantly negatively correlated with parental hostility. (4) Conclusions: Since there is a positive correlation between children’s aggression and self-control, it is necessary to develop strategies to improve self-control when seeking intervention measures for children’s aggression. In addition, since there is a significant quantitative correlation between children’s aggression and parental hostility, it is necessary to deal with parental hostility in order to control children’s aggression. Since there is a significant negative correlation between parental hostility and children’s self-control, it is necessary to emphasize the importance of parental attitudes and behaviors to improve children’s self-control.

## 1. Introduction

As the Ministry of Education reported in 2018, children of school age were more likely to experience being a school violence victim or perpetrator than adolescents [1]. As revealed by Statistics Korea in 2018, children of school age were more likely to be a victim of physical aggression and were at least twice as likely to be bullied as adolescents [2].

The experience of being a school violence victim can make children aggressive [3,4]; such aggression may not be easily reduced as they grow up but can sometimes be expressed through more aggressive behavior and can develop into criminal behavior when neglected [5]. In addition, as children’s aggression increases, their immersion in games increases, increasing the risk of game addiction [6]. Furthermore, the higher the aggression, the greater the increase in school-violence-perpetrating behavior [7]. As such, children’s aggression can develop into individual problem behaviors beyond aggression, so it is necessary to pay attention to it. Since aggression in childhood is deeply associated with that in adulthood [8], addressing aggression in childhood is critical.

Children’s aggression includes overt aggression, involving physical and verbal aggression, and relational aggression, which means collective and indirect aggression that damages peer relationships through social relationships, such as intentionally bullying individuals using group power or pressure or spreading bad rumors [9]. In the meantime, research on aggression has focused on overt aggression, which is common in boys, and has focused on finding and mediating related variables. However, it has emerged that relational aggression, which can damage peer relationships without outward aggression, should also be considered along with overt aggression [10]. Although relational aggression is more common in girls, it can appear in both sexes as children grow older [11]. These results revealed that, when children’s aggression is studied only with overt aggression, the relational aggression experienced by a significant number of children is excluded from the aggressive group, and as a result, it is difficult to accurately identify children’s aggression. In particular, most previous studies on reducing aggression through interventions measured and reviewed the effect of only overt aggression [12,13]. However, since both overt aggression and relational aggression cause different maladaptive outcomes [11], interventions aimed at reducing aggression need to address both forms of aggression together.

From a psychoanalytical perspective, Freud considered sexual desire as well as aggression as the most fundamental human drive impulses [14]. Children’s self-control, as the ability to control and regulate such an impulse, can be regarded as a factor related to their aggression [15]. Self-control refers to an internal motivation to change behavior for a positive outcome. Education to improve self-control can reinforce an individual’s problem-solving abilities [16]. In case of a failure to regulate the impulse to be aggressive, aggression can be reduced through self-control [17] and be regulated by improving self-control [18]. However, in previous studies [16,17,18], self-control improvement programs were applied that did not consider various aspects of aggression. They considered only one aspect of aggression and had limitations in being selected as an intervention method to reduce both overt and relational aggression. Therefore, it is expected that this study will provide basic data for the development of interventions to improve self-control suitable for aggression improvement programs by confirming the relevance of general aggression, including children’s overt and relational aggression, to children’s self-control.

The behavioral and cognitive changes found in observations of a specific model are crucial factors that cause aggression in childhood, and children consciously and unconsciously resemble their parents when they spend much time together [19]. Aggression, caused by negative emotions, such as anger, in individuals full of hostility, refers to the socially undesirable behavior of intentionally threatening others [20]. As a cognitive factor causing emotional problems in individuals, hostile automatic thought, which can represent hostility, refers to having a pessimistic and cynical view of others and the world in terms of anger, and it motivates aggressive behavior to harm or destroy others [21]. Individuals with hostile automatic thoughts are more likely to become angry. Anger is one of the determinants of aggression, and a sense of anger is considered to cause aggressive behavior [22]. In addition, it was found that the more hostile thinking an individual experiences, the more aggressive behavior they exhibit and the more helplessness they experience, making adaptive coping more difficult in problem-solving situations [23]. Since hostility is a cognitive aspect of aggression, [24] it can be said that a parent’s hostile automatic thought, possibly used to assess a hostile attitude, is strongly associated with a child’s aggression.

In addition, in previous studies, the more positive the parenting attitude, the higher the level of self-control of the child [25], and the more hostile the parent’s parenting attitude, the lower the level of self-control of the child [26]. It was also found that parental verbal abuse had a significant effect on children’s self-control [27]. Parents’ attitudes toward their child gradually increase the child’s self-control and have a very large effect on the individual [26].

Many studies have been conducted on the relationship and influencing factors between parents’ rearing attitudes and children’s aggression and self-control. In particular, children are naturally influenced by their parents, with whom they spend the most significant amount of time [19]. In other words, this means that children can learn not only the general behaviors of the parents who care for them, but also all the behaviors and attitudes that the parents show in everyday life. As such, since children can acquire parents’ attitudes consciously and unconsciously, it is necessary to examine the relationship among parents’ hostility toward others, children’s aggression, and children’s self-control. 

However, little research has been conducted on the association between parental hostility and a child’s self-control and aggression. Therefore, this research aims to provide basic data for research on the development of interventions for children’s aggression by identifying parental hostility, children’s self-control and aggression, and variables related to children’s aggression.

The specific objectives are as follows:

(1) To identify aggression based on the general characteristics of the child;
(2) To investigate parental hostility and a child’s self-control and aggression;
(3) To determine the correlation between parental hostility and a child’s self-control and aggression.

## 2. Materials and Methods

### 2.1. Research Design

This study was a descriptive study in which data were collected in a self-reported way to obtain information on the correlations between variables, and a descriptive correlational design was used to define the relationships between variables.

### 2.2. Subjects

The subjects of this study were obtained through the convenience sampling of elementary school students and their parents living in C, K Provinces and D cities as an accessible population. We visited three schools and, with the permission of teachers and students at the schools, sent home questionnaires for parents and children and consent forms for participation in the study. The questionnaires for parents who responded to the questionnaire in hopes of participating in the study were sealed and collected. Only children whose parents consented to their children’s participation in the study were asked to complete the children’s questionnaire. The specific subject selection criteria are 5th- or 6th-grade students attending a general elementary school, students who understand and agree to the purpose of the study, and their fathers or mothers. Parents and children who agreed to the purpose of this study and accepted participation were selected as subjects through convenience sampling. The sample size was estimated using the G*Power 3.1.9.2 program. For the correlation analysis, the G*Power program calculated a median effect size of 0.30, a significance level of 0.05, and a power of 0.95, resulting in 134 persons. One hundred and seventy-four questionnaires were distributed, taking the dropout rate into account, both because the respondents were elementary school children and because data from their parents were also needed. One hundred and sixty-four copies were used for the analysis, excluding ten copies without responses among the collected questionnaires.

### 2.3. Instruments

#### 2.3.1. Parental Hostility

Hostility refers to an attitude of seeing others or the world in a negative and cynical way [28]. The hostile automatic thought scale—Korean version (HAT-K), which is Seo’s validated version of the hostile automatic thought scale (HAT) [21] developed by Snyder et al. and targets Korean adults—was used to measure parental hostility by asking parents questions in person [23]. It is composed of 30 items: 11 for physical aggression, 10 for derogation, and 9 for revenge. Each item is rated from 1 (“not at all”) to 5 (“very so”) on a Likert scale, with a higher score meaning a more hostile attitude toward others. As for the reliability in Seo’s study [23], Cronbach’s α was estimated at 0.96 for physical aggression, 0.89 for derogation, 0.91 for retaliation, and 0.91 for hostility in general. As for the reliability in this study, Cronbach’s α was estimated at 0.946 for physical aggression, 0.948 for derogation, 0.951 for retaliation, and 0.975 for hostility in general.

#### 2.3.2. Child Aggression

Aggression is physical and verbal aggression toward others, and it includes indirect forms of relational aggression that cause harm to others by damaging or threatening an individual’s feelings or relationships [10]. In this study, children’s aggression was assessed by having the children themselves respond to a questionnaire. The scale by Ha and Kim was used to measure a child’s aggression [29]. This tool is composed of 16 items: 4 for proactive overt aggression, 4 for proactive relational aggression, 4 for reactive overt aggression, and 4 for reactive relational aggression. Each item is rated from 1 (“not at all”) to 5 (“very so”) on a Likert scale, with a higher score meaning more aggressive. As for the reliability in Ha and Kim’s study [29], Cronbach’s α was estimated at 0.81 for proactive overt aggression, 0.81 for proactive relational aggression, 0.81 for reactive overt aggression, 0.82 for reactive relational aggression, and 0.93 for aggression in general. As for the reliability in this study, Cronbach’s α was estimated at 0.734 for proactive overt aggression, 0.645 for proactive relational aggression, 0.793 for reactive overt aggression, 0.706 for reactive relational aggression, and 0.886 for aggression in general.

#### 2.3.3. Child Self-Control

Self-control is the ability to control and endure an individual’s temporary satisfaction and impulses, and the means to control one’s cognition, emotion, and behavior as desired [30]. In this study, children’s self-control was assessed by having the children themselves respond to a questionnaire. The self-control scale by Nam and Ok was used to measure self-control [31]. This scale is composed of 20 items: 10 for pursuing long-term satisfaction and 10 for pursuing immediate satisfaction, such as being impulsive and self-centered or acting before saying. The response to each question is a Likert scale ranging from 1 point for “not at all” to 5 points for “very much so”, and the higher the sum of the scores, the higher the self-control. In the study by Nam and Ok [31], Cronbach’s α was 0.706 for self-control in general, and in this study, Cronbach’s α was estimated at 0.834 for long-term satisfaction, 0.800 for immediate satisfaction, and 0.879 for self-control in general.

### 2.4. Data Collection

Data collection was performed from April to May 2019. Before the survey, the school principals, class teachers, and health teachers explained the study’s content and purpose and asked for help. To obtain consent from parents for their children’s participation in the research, a description of the purpose and methods of the study, the anonymity and autonomy of research participation, the possible advantages and disadvantages, and the possibility of withdrawing from the research was sent home, and written consent was obtained from the parents. The questionnaires for parents were sent home so they could complete them; then, they were sealed and collected. Questionnaires were distributed only to children whose parents agreed to participate in the study. The participants were asked to complete a questionnaire personally and were given a small gift.

### 2.5. Data Analysis

The collected data were analyzed using the SPSS 25.0 program. The respondents’ general characteristics were analyzed using descriptive statistics. Parental hostility and the children’s self-control and aggression were analyzed using descriptive statistics involving the mean and standard deviation. The correlation between parental hostility and a child’s self-control and aggression was analyzed using Pearson’s correlation coefficient. Cronbach’s alpha value was calculated to identify the reliability of the internal consistency of the measurement tool.

### 2.6. Ethical Considerations

Before data collection, this study obtained approval (EU18-106) from the Institutional Review Board of E university in D metropolitan city in South Korea. Before conducting the survey, the principal, homeroom teacher, and health teacher of the school where the survey was to be conducted were introduced to the research contents and purpose, and their cooperation was sought. To collect data, the questionnaires for children were only distributed in those cases where both the parents and their children agreed to participate in the research. Researchers and research assistants entered the classroom where the children whose parents agreed to participate in the study were, explained to the students about the content and purpose of the study, the contents of the questionnaire, and guaranteeing privacy, and then allowed the children to respond directly. For confidentiality reasons, the questionnaires were sealed immediately after completion. The participants were given an oral or written explanation that they could withdraw from the research at any time. They were notified that the collected data would be kept locked safely and that the research materials would be kept for three years after the study’s end and mechanically destroyed afterward. 

## 3. Results

### 3.1. Participants’ General Characteristics

The participants’ general characteristics—children and their parents—are presented in Table 1. Of the children participating in the research, 139 (84.8%) were fifth graders and 25 (15.2%) were sixth graders, and 91 (55.5%) were boys and 73 (44.5%) were girls. As for birth order, 70 children (42.7%) were the oldest, 64 (39.0%) were the second oldest, 14 (8.5%) were the third oldest or more, and 16 (9.8%) were only children. As for the perceived household economic level, 80 (48.8%) were at the high level, 79 (48.2%) were at the middle level, and 5 (3.0%) were at the low level. 

Of the fathers participating in the research, 11 (6.7%) were in their thirties, 135 (82.3%) were in their forties, and 18 (11.0%) were in their fifties or older. Of the mothers, 40 (24.4%) were in their thirties, 114 (69.5%) were in their forties, and 10 (6.1%) were in their fifties or older. As for the fathers’ education, 46 (28.0%) were high school graduates or lower, 23 (14.0%) were college graduates, 67 (40.9%) were university graduates, and 28 (17.1%) were graduate school graduates. As for the mothers’ education, 46 (28.0%) were high school graduates or lower, 29 (17.7%) were college graduates, 59 (36.0%) were university graduates, and 30 (18.3%) were graduate school graduates. As for the average monthly household income, 15 households (9.1%) earned not more than KRW 2.89 million, 31 (18.9%) earned KRW 2.9 million to 3.99 million, 41 (25.0%) earned KRW 4 million to 5.19 million, and 77 (47.0%) earned at least KRW 5.2 million (Table 1).

### 3.2. Aggression of the Participants (Children)

The differences in aggression according to the general characteristics of children were as follows (Table 2). Aggression was scored at 19.14 ± 4.71 points for boys and 17.93 ± 3.53 points for girls, showing a significant difference (*t* = 3.081, *p* = 0.004). However, there were no significant differences in aggression according to grade, birth order, or economic status.

### 3.3. Mean Score of Child Aggression, Self-Control, and Parental Hostility

The levels of aggression, self-control, and parental hostility of children in this study were as follows (Table 3).

(1) Child Aggression
  The children’s aggression was analyzed using the total score, and the total average was 18.60 ± 4.26. Among the sub-items, they scored 4.59 ± 1.17 for proactive overt aggression, 4.37 ± 0.95 for proactive relational aggression, 5.04 ± 1.73 for reactive overt aggression, and 4.60 ± 1.29 for reactive relational aggression.
(2) Child Self-Control
  The children’s self-control was analyzed using the total score, and the total average of the children’s self-control was 77.78 ± 11.51.
(3) Parental Hostility
  Parental hostility was analyzed using the total score, and the total average was 64.71 ± 24.15. Among the sub-items, they scored 19.71 ± 8.15 for physical aggression, 25.96 ± 9.69 for derogation, and 19.04 ± 7.97 for retaliation (Table 3).

### 3.4. Correlation among Child Aggression, Self-Control, and Parental Hostility

In this study, the results of analyzing children’s aggression, self-control, and parental hostility using a correlation analysis were as follows (Table 4). The children’s aggression was positively correlated with parental hostility (r = 0.172, *p* = 0.027) and was also positively correlated with such sub-factors of hostility as physical aggression (r = 0.171, *p* = 0.028) and retaliation (r = 0.171, *p* = 0.029). The children’s proactive relational aggression, a sub-factor of aggression, was positively correlated with physical aggression, a sub-factor of parental hostility (r = 0.160, *p* = 0.041). The children’s reactive relational aggression, a sub-factor of aggression, was positively correlated with parental hostility (r = 0.162, *p* = 0.038) and retaliation, which is its sub-factor (r = 0.183, *p* = 0.019).

A child’s self-control was significantly negatively correlated with aggression (r = −0.484, *p* < 0.001) and was negatively correlated with such sub-factors of aggression as proactive overt aggression (r = −0.382, *p* < 0.001), proactive relational aggression (r = −0.327, *p* < 0.001), reactive overt aggression (r = −0.435, *p* < 0.001), and reactive relational aggression (r = −0.430, *p* < 0.001). The children’s self-control was significantly negatively correlated with parental hostility (r = −0.215, *p* = 0.006) and was negatively correlated with physical aggression (r = −0.225, *p* = 0.004), derogation (r = −0.158, *p* = 0.043), and retaliation (r = −0.230, *p* = 0.003), among its sub-factors.

Pearson’s correlation analysis expresses the strength of a linear relationship between two continuous variables as a single value, the correlation coefficient (r). As the correlation coefficient r approaches 1, the correlation between the two variables becomes more pronounced as a positive correlation, close to a straight line, and as it approaches −1, the correlation between the two variables becomes more pronounced as a negative correlation. A recent study reported that the traditional Cohen’s guideline for the correlation coefficient r was too stringent [32]. The new criterion recommended by Funder and Ozer is that an effect size r of 0.10 represents a small but potentially more ultimate effect, an effect size of 0.20 is somewhat explanatory and practically useful even in the short term, and an effect size r of a value of 0.30 represents a large and potentially powerful effect in both the short and long term. In addition, it is reported that an effect size r of 0.40 or higher is highly likely to be an overestimation that is rarely found in large samples or replications. According to the criteria of Funder and Ozer, the interpretation of the correlation coefficient r in this study can be mostly explained (Table 4).

## 4. Discussion

In this study, male aggression was higher than female aggression. In a previous study conducted on elementary school students [33,34], the results indicating that male aggression was high support this study. This is thought to be due to the social and cultural background, in which Korean society is more tolerant of aggressive behavior from boys than girls and emphasizes femininity to girls. The sociocultural background is one of the main factors that causes boys to engage in aggressive behavior [35]. Therefore, more attention is needed on boys’ aggression in interventions to control children’s aggression.

In this study, children’s aggression was significantly negatively correlated with their self-control: the better at self-control children were, the less likely they were to manifest aggressive behavior. Children that are good at self-control can control their cognition, emotion, and behavior as needed and behave according to the pursuit of long-term satisfaction [36]. In other words, children that are good at self-control can control aggressive behavior to achieve better outcomes instead of aggressively coping in a maladaptive way with a situation. In addition, children’s self-control was found to be an important factor that can control the development of problematic behaviors in children with high aggression [37]. Aggression in the form of intentionally excluding others from a group or bullying others is highly related to a child’s low self-control [38]. Significantly, this study measured the four factors of aggression from various angles by combining the two dimensions of overt–relational forms and reactive–initiative mechanisms, and it found that general aggression had a deep negative correlation with children’s self-control. Therefore, it is thought that this study provides basic data for devising a more appropriate self-control training program when seeking intervention measures for children’s aggression.

In this study, parents’ hostility was significantly positively correlated with a child’s aggression: the more hostile an attitude parents had toward others, the more aggressive their children were. This result is supported by the finding of a previous study that, because children adopt the same damaging and rejecting attitude and behavior as their parents as a strategy for resolving a problematic situation, such behavior can be expressed in a disposition of aggression toward others [39]. Children who experienced or witnessed their parents’ aggressive behavior while they grew up were more aggressive and more likely to show aggressive behavior [40]. Aggressive behavior results from a hostile awareness of others’ intentions [41], and a parent’s hostile automatic thought is associated with the child’s aggression. It is, therefore, necessary to address parental hostility to control a child’s aggression. To children, parents are like the universe. Children see and learn all the actions of their parents consciously or unconsciously in everyday life. In unexpected situations that occur in the daily lives of parents and children, children become more sensitive and intensely watch and learn from their parents’ behavior. At this time, it is very important for the child to reduce the behaviors that show hostile thoughts, to not express emotions in an undesirable way even in situations where such emotions arise, to refrain from negative emotions, and to show desirable gentleness.

Parents’ hostility was significantly negatively correlated with a child’s self-control: the more hostile an attitude parents had toward others, the poorer their children were at self-control. This result is similar to the finding that children who experienced abuse based on their parents’ aggressive behavior can experience a negative effect on their self-control, being poorer at impulse control, as they act before they say and are less capable of enduring frustration or failure [42]. In addition, in previous studies, the parents’ aggressive behavior, including the abuse or neglect of their children, was related to their children’s low self-control [43]. This finding implies that children constantly exposed to their parents’ aggressive behavior can have difficulty developing self-control. When parents are affectionate, give psychological stability, and become a self-control model for their children, the children can more easily develop self-control [44]. It is, therefore, necessary to emphasize the importance of parental attitudes and behavior in pursuit of a child’s self-control. 

Most prior studies were conducted on the association between a sense of hostility and the disbelief that parents showed when they reared their children and their children exhibited aggression. In contrast, this study investigated the correlation between the parents’ sense of aggression, derogation, and retaliation against others and their children’s aggression and self-control. It confirmed that the parents’ hostile attitude toward others was significantly positively correlated with their child’s aggression and was significantly negatively correlated with their child’s self-control. This implies that a parent’s daily attitude toward others and childrearing can affect a child’s aggressive behavior, attitude, and self-control. It is, therefore, necessary to develop an intervention, by considering parental attitude and behavior, to control children’s aggression and improve their self-control.

### Limitations

Since this study was conducted on fifth and sixth graders of elementary schools in some regions, it is difficult to generalize to the entire group of children. Therefore, follow-up studies should consider the scope of the study subject in various ways. In addition, among various factors related to children’s aggression, only the relationship between parental hostility and children’s self-control was confirmed. Therefore, further studies on various factors related to children’s aggression are needed. In this study, children’s aggression was collected through a structured questionnaire. While multiple-choice questionnaires have strengths in collecting information on the direction and intensity of respondents’ opinions and attitudes, inexperienced children may select responses incorrectly, leading researchers to misinterpret research findings. Therefore, it will be necessary to diversify the measurement strategies for the variables in follow-up studies.

## 5. Conclusions

This descriptive research aimed to investigate children’s aggression and self-control and parental hostility, analyze the correlations between them, and provide basic data that could help reduce children’s aggression and promote their healthy growth and development. In this study, boys’ aggression was higher than girls’ aggression. The better children were at self-control, the less aggressive they were. The more hostile an attitude parents had toward others, the more aggressive and poorer at self-control their children were. Therefore, when planning interventions to prevent or control children’s aggression, children’s self-control and parents’ attitudes should be considered. In addition, it is necessary to recognize that there is a difference in aggression according to the gender of the child, and to design a program that considers gender.

Based on the above research results, the following suggestions are made. First, since this study was collected before the outbreak of COVID-19, it may be limited in explaining the current situation. Therefore, a repeat study is needed in subjects who have experienced COVID-19. Second, since this study targeted elementary school students, it is expected that a follow-up study on middle and high school students together to see how the problems related to aggression latent in childhood are revealed in adolescence will be meaningful. Third, since this study revealed the relationship among children’s aggression, self-control, and parental hostility, we suggest a mediation analysis study on the factors affecting children’s aggression. Fourth, in this study, the correlation coefficient r of children’s aggression and self-control was −0.484, indicating the large effect size suggested by Funder and Ozer, so it can be considered an excessive estimate. Therefore, a repeat study with a larger sample size is recommended.

## Figures and Tables

**Table 1:** General characteristics of participants.

			(N = 164)
Participants	Variables	Categories	N (%)
Child	Grade	5	139 (84.8)
		6	25 (15.2)
	Gender	Male	91 (55.5)
		Female	73 (44.5)
	Birth order	1	70 (42.7)
		2	64 (39.0)
		≥3	14 (8.5)
		Only child	16 (9.8)
	Economic status	High	80 (48.8)
		Middle	79 (48.2)
		Low	5 (3.0)
Parent	Father’s age (year)	30–39	11 (6.7)
		40–49	135 (82.3)
		≥50	18 (11.0)
	Mother’s age (year)	30–39	40 (24.4)
		40–49	114 (69.5)
		≥50	10 (6.1)
	Father’s education level	Less than high school	46 (28.0)
		Diploma	23 (14.0)
		Bachelor	67 (40.9)
		Graduate school	28 (17.1)
	Mother’s education level	Less than high school	46 (28.0)
		Diploma	29 (17.7)
		Bachelor	59 (36.0)
		Graduate school	30 (18.3)
	Family income (KRW ten thousand)/month	≤289	15 (9.1)
		290–399	31 (18.9)
		400–519	41 (25.0)
		≥520	77 (47.0)

**Table 2:** Aggression of the participants (children).

				(N = 164)		
Participants	Variables	Categories	N (%)	M ± SD	*t* or F (*p*)	Effect Size (*d*) or (*η*^2^)
Child	Grade	5	139 (84.8)	18.67 ± 4.37	0.463 (0.644)	0.107
		6	25 (15.2)	18.24 ± 3.60		
	Gender	Male	91 (55.5)	19.14 ± 4.71	3.081 (0.004)	0.291
		Female	73 (44.5)	17.93 ± 3.53		
	Birth order	1	70 (42.7)	18.83 ± 4.32	1.134 (0.337)	0.202
		2	64 (39.0)	18.05 ± 3.75		
		≥3	14 (8.5)	20.50 ± 6.55		
		Only child	16 (9.8)	18.00 ± 3.37		
	Economic status	Low	5 (3.0)	18.20 ± 3.83	0.809 (0.447)	0.101
		Middle	79 (48.2)	18.19 ± 3.55		
		High	80 (48.8)	19.04 ± 4.88		

**Table 3:** Mean score of child aggression, self-control, and parental hostility.

					(N = 164)
Participants	Variables	Item	Range	Mean	SD
Child	Aggression	16	4~64	18.60	4.26
	Proactive overt	4	4~16	4.59	1.17
	Proactive relational	4	4~16	4.37	0.95
	Reactive overt	4	4~16	5.04	1.73
	Reactive relational	4	4~16	4.60	1.29
	Self-control	20	20~100	77.78	11.51
Parent	Hostility	30	30~150	64.71	24.15
	Physical aggression	11	11~55	19.71	8.15
	Derogation	10	10~50	25.96	9.69
	Revenge	9	9~45	19.04	7.97

**Table 4:** Correlations among variables.

									(N = 164)	
Variable	1.	1.1.	1.2.	1.3.	1.4.	2.	2.1.	2.2.	2.3.	3.
	r(*p*)	r(*p*)	r(*p*)	r(*p*)	r(*p*)	r(*p*)	r(*p*)	r(*p*)	r(*p*)	r(*p*)
1. Child Aggression	1									
								
1.1. Proactive overt	0.844 ***	1								
	(0.000)									
1.2. Proactive relational	0.805 ***	0.633 ***	1							
	(0.000)	(0.000)								
1.3. Reactive overt	0.877 ***	0.706 ***	0.556 **	1						
	(0.000)	(0.000)	(0.000)							
1.4. Reactive relational	0.771 ***	0.469 ***	0.606 ***	0.505 ***	1					
	(0.000)	(0.000)	(0.000)	(0.000)						
2. Parental Hostility	0.172 *	0.148	0.143	0.125	0.162 *	1				
	(0.027)	(0.058)	(0.067)	(0.112)	(0.038)					
2.1. Physical aggression	0.171 *	0.128	0.160 *	0.140	0.145	0.918 ***	1			
	(0.028)	(0.103)	(0.041)	(0.074)	(0.064)	(0.000)				
2.2. Derogation	0.145	0.146	0.099	0.105	0.132	0.927 ***	0.729 ***	1		
	(0.065)	(0.062)	(0.206)	(0.183)	(0.092)	(0.000)	(0.000)			
2.3. Revenge	0.171 *	0.141	0.150	0.107	0.183 *	0.965 ***	0.872 ***	0.849 ***	1	
	(0.029)	(0.072)	(0.055)	(0.171)	(0.019)	(0.000)	(0.000)	(0.000)		
3. Child Self-Control	−0.484 ***	−0.382 ***	−0.327 ***	−0.435 ***	−0.430 ***	−0.215 **	−0.225 **	−0.158 *	−0.230 **	1
	(0.000)	(0.000)	(0.000)	(0.000)	(0.000)	(0.006)	(0.004)	(0.043)	(0.003)	

* *p* < 0.05; ** *p* < 0.01; *** *p* < 0.001.

## Data Availability

The data used are confidential, and the study participants have not consented to data sharing. Due to the sensitive nature of the personal information asked for by the questions in this study, the survey respondents were assured that the raw data would be kept confidential and would not be shared.
